# Notes on fiber length measurements: A case study in the underbelly of open source neuroscience

**DOI:** 10.1016/j.neuroimage.2022.119738

**Published:** 2022-12-01

**Authors:** Claude J Bajada, Robert E Smith, Svenja Caspers

**Affiliations:** a Department of Physiology and Biochemistry, Faculty of Medicine and Surgery, University of Malta, Msida, Malta; b University of Malta MRI Research Platform (UMRI), University of Malta, Msida, Malta; c The Florey Institute of Neuroscience and Mental Health, Melbourne Brain Centre - Austin Campus, Heidelberg, VIC 3084, Australia; d Florey Department of Neuroscience and Mental Health, The University of Melbourne, Parkville, VIC 3010, Australia; e Institute of Neuroscience and Medicine (INM-1), Research Centre Juelich, 52425 Juelich, Germany; f Institute for Anatomy I, Medical Faculty & University Hospital Duesseldorf, Heinrich-Heine-University Duesseldorf, 40221 Düsseldorf, Germany

**Keywords:** Open science, Software bug, Tractography

## Abstract

Being on the bleeding edge of research requires the use of new and regularly updated software. The result is the occasional and inevitable occurrence of bugs. In the following work we present a case study where a feature request introduced a bug in a neuroimaging software package, which had consequences for the quality of results in a published article. We discuss the process of diagnosis, rectification and analysis replication.

## Highlights

• We present a case study where a feature request introduced a bug in a neuroimaging software package.
• We discuss the process of diagnosis, rectification and analysis replication.
• We describe the implications of the process on the open science publishing.

## Introduction

The price of being on the bleeding edge of research, with new and regularly updated software, is the occasional yet inevitable occurrence of bugs. Such errors are not limited to code used only within one's own lab (Miller, 2006; Strand, 2020), but have also been found in standard, long established software suites (Eickhoff et al., 2017; Eklund et al., 2016), where even minor bugs can have large downstream effects upon the body of literature.

In the following commentary we present a case study where a feature request introduced a bug in a neuroimaging software package, which had consequences for the quality of results in a published article. This article serves multiple purposes in this context:

1 To satisfy the obligation of publishing a corrigendum regarding the affected article.
2 To provide full details of the bug itself and its influence on the published results in the interest of scientific transparency.
3 To reference public communications, software changes, and corresponding software engineering practices, in the hope of serving as an exemplar for the processes necessary to uphold the tenets of open science in the context of scientific software usage.

## Summary of affected study

Our case study relates to the work done in Bajada et al. (2019) (“Fiber length profiling: A novel approach to structural brain organization”). This work examined the distributions of lengths of white matter connections in 76 individuals from the healthy adult WU-Minn Human Connectome Project (HCP) dataset, mapping the lengths of diffusion tractography streamlines across the cortical surface across individuals. Streamlines were initialized individually from each cortical surface vertex, spatially normalized to a standard space, and histograms of the lengths of streamlines at each vertex were produced. For each vertex, each bin of the streamline length histogram corresponded to a map of streamlines within a narrow range of lengths; these maps were then correlated with various structural and functional metrics.

## Issue description

The study used the *MRtrix3* software package ((Tournier et al., 2019); www.mrtrix.org) to perform streamline tractography, transform streamlines to standard space, and compute streamline lengths; version 3.0_RC3 was used. The *MRtrix3* function tckstats is responsible for computing streamline lengths of a pre-generated tractogram.

**Fig. 1:**
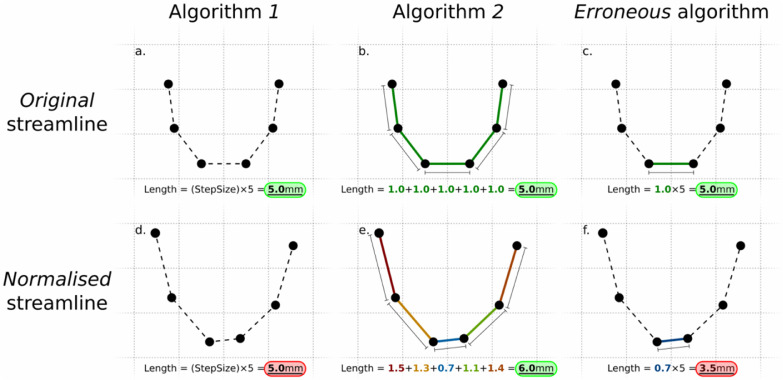
Demonstration of various algorithms (columns) used to calculate streamline length for: (top row) generated streamline with fixed step size; (bottom row) non-linearly transformed streamline. Dashed lines indicate that inter-vertex distance is assumed; solid lines indicate explicit distance calculation.
  When all adjacent vertices are known to be equidistant (i.e. the tractography “step size” is constant), this is highly computationally efficient.
2 The sum of the distances between adjacent vertices (Fig. 1b). No assumption regarding consistency of these distances is made in this case.

The specific issue on which we report here relates to the calculation of streamline lengths. Each streamline is simply an ordered set of vertices in 3D space. While instinctively trivial, there are a number of ways in which the length of a streamline may be calculated. Two possible algorithms are:

1 The product of the fixed distance between vertices (the so-called “step size” of the tracking algorithm) and one less than the number of vertices (Fig. 1a).

Because this particular study involved a *non-linear* spatial transformation of streamline data to a standard space, which has the potential to modulate the distances between streamline vertices (Fig. 1 bottom row), a function implementing algorithm 2 was required in this context; this was nominally achieved using command-line option -explicit in command tckstats, previously implemented by author RS upon request from author CB.

Following acceptance of the case study article, CB commenced work on a related project, involving *extracting* streamlines from a tractogram based on their length, but where streamline lengths needed to be calculated using algorithm 2 above (as the streamlines had been spatially, non-linearly, normalized). As streamline extraction based on length using *MRtrix3* command tckedit operated via algorithm 1 only, CB utilized a workaround whereby each streamline would be first *resampled* to a set of equidistant vertices based on a continuous spline representation (*MRtrix3* command tckresample), such that their lengths would then be appropriately quantified by algorithm 1.

CB discovered a discrepancy between the lengths of these explicitly resampled streamlines using algorithm 1, and the lengths of the streamlines that had been spatially normalized *but not resampled*, which should nominally have been performed using algorithm 2. This information was reported by CB to the developers of the *MRtrix3* software package via the relevant online community forum. Upon investigation by RS it was discovered that command tckstats was in fact *not* utilizing algorithm 2 when explicitly requested. It was instead using a function intended for the case where step size information was not available from metadata, yet was *nevertheless constant*: it would infer the fixed step size based on the distance between the two central vertices of the streamline, and subsequently utilize algorithm 1 (Fig. 1c). For streamlines where the distance between adjacent vertices varies along their length—for instance, those that have undergone a non-linear spatial transformation—this yields an *incorrect answer* (Fig. 1f).

## Issue rectification

Complete resolution of such an issue requires redress in multiple domains. Here we summarize the steps taken in light of this particular discovery.

### Registration of issue on GitHub and bug rectification

RS created Issue #1501 on the GitHub repository of the *MRtrix3* software, describing the fault and initiating more developer-centric dialogue. The fault itself was rectified by RS in git commit bbfdaeaa, altering the behavior of the relevant function to that of algorithm 2 described above. Pull request #1510 was then opened, indicating the desire to propagate this change to the git “master” branch (the default access point upon downloading the code). Upon completion of Continuous Integration (CI) testing and compulsory independent code review, this Pull Request was completed via merge commit d7f497c9. The rectified code was thereby available to any existing user upon an explicit code update to their local master branch, and would be automatically included by default for any new clone of the software code.

(While staged on the master branch in preparation for inclusion in a future software patch, this fix was eventually superseded by deeper software changes—see below—that were included in the *MRtrix3* production release 3.0.0, for which user compilation from source code was no longer necessary)

### Announcement of fix

Both the underlying issue and availability of the resulting fix were publicly announced on the community forum.

### Re-processing study data

**Fig. 2:**
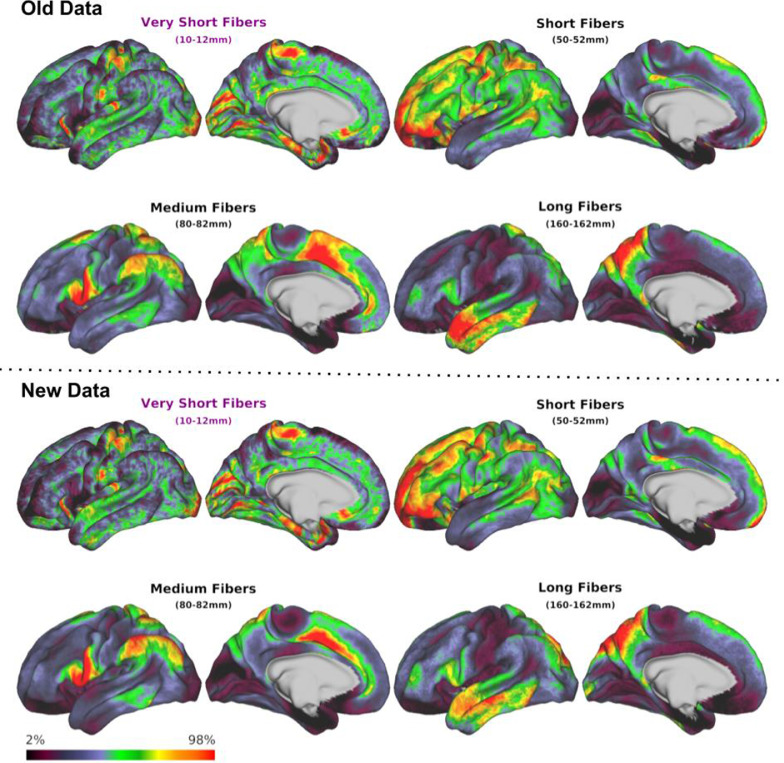
Changes in experimental outcomes as a consequence of software revision. Top: Original results as reported in (Bajada et al., 2019), which made use of the erroneous streamline length calculation algorithm; bottom: revised results obtained with proper use of algorithm 2. The heat maps represent the relative density of streamlines across the cortex at a given length, where 100% represents the maximum streamline count at a particular length (see Figs. 1, 4 and 5 in the original publication).

**Fig. 3:**
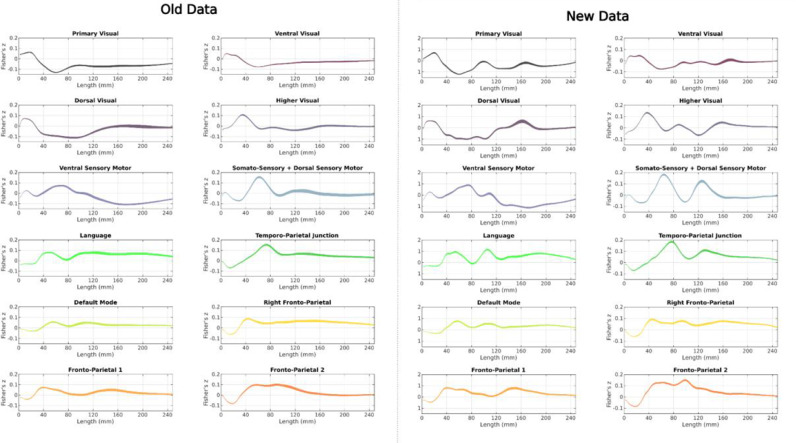
Changes in experimental outcomes as a consequence of software revision. Left: Original results as reported in (Bajada et al., 2019), which made use of the erroneous streamline length calculation algorithm. Functional profiles of length maps across a range of lengths (2–250 mm) correlated with 12 different resting state functional maps; line thickness indicates the 95% confidence interval of the mean Fisher's Z transformed correlation score. Right: Recalculated results using rectified streamline length calculation algorithm.

Following the fix being announced, the original data were reanalyzed with the updated algorithm. The new results are displayed in comparison to those originally reported in Figs. 2 and 3. Overall experimental observations and conclusions were relatively unaffected, and correlations between streamline length maps and functional information (Fig. 3 right) show considerably greater contrast (ie. graphs are more unique across different networks and possess peaks of greater magnitude) in those results generated with the revised length calculation algorithm, suggesting that some of this information was obscured in the originally reported results by the influence of the erroneous algorithm.

### Updating study data online

Completion of the original study included the public distribution of not only figures, but data encoding summary statistics of streamline lengths in voxel and surface formats. These data were reproduced using the fixed code and uploaded to a new BALSA repository with the description of the original repository providing the link to the new one.

### Readership notification

In circumstances where an error in published scientific work necessitates correction, an erratum is typically published stand-alone, very briefly describing the nature of the fault (though original published articles typically remain unaltered). Here, not only is the designation of “erratum” arguably not applicable as the error was the fault of neither author nor publisher, this case study supersedes such notification.

### Deeper software changes

This rectification further highlighted how one erroneous assumption can have unforeseen ramifications elsewhere within a software package. A corresponding set of wider software changes were deemed appropriate for a subsequent larger software update to ensure that intended criteria regarding streamline lengths would *always* be accurately satisfied, even in the presence of confounds such as:

• Streamline downsampling following tracking (used to reduce file size).
• Arbitrary resampling of streamline vertices (e.g. *MRtrix3* command tckresample).
• Truncation of the final streamline segment (Smith et al., 2012).
• Non-linear spatial transformation (as in “Issue description”).

These technical changes were described extensively in Pull Request #1507. Following the established software engineering pipeline, these changes were *not* immediately merged to the change to the master branch, due to modifying empirical command behavior in a non-critical fashion. They were instead merged to a central development code branch for eventual inclusion in the next software update. This corresponded to the *MRtrix3* production release 3.0.0, where these changes were described in the changelog (note that those changes described in “Registration of issue on GitHub and bug rectification” above were superseded by such). While these changes are beyond the scope of the specific issue affecting the original article, detail is nevertheless provided here to exemplify how identification of erroneous assumptions can have ramifications for aspects of software behavior beyond the original observation.

## Discussion

### Software in open science

This short case report highlights the difficult realities faced in scientific research relying on bleeding-edge software packages that are themselves continuous works in progress. It was crucial in identifying and rectifying this issue that the software in question is *both* open-source *and* has open online dialogue available with responsive developers. The former receives deserved attention in modern science, and facilitates independent isolation of causative factors where errors or differences are observed. For instance, if one discovers changes in outcomes across software versions, in the open source case (eg. as has occurred for the popular FreeSurfer package (Fischl, 2012); for instance (Gronenschild et al., 2012)) researchers can independently investigate the underlying causative factor(s), whereas in the closed source case (eg. (Lefebvre et al., 2018)) this typically is hardly possible. Here we postulate that the latter aspect—open online dialogue—warrants comparable prioritization in the research software domain, as this is what obliges a response in such scenarios that is transparent, proportional, and immediately discoverable to other potentially affected parties. Indeed this article is a mutual effort between the first author of the affected article and the developer responsible for the underlying issue, and includes hyperlinks to the full history of issue rectification, in the hope of serving as an exemplar for such relationships and augmenting existing precedent for dealing with such situations (Eickhoff et al., 2017).

### Dynamic scientific publication

While there are established software engineering practices for version control that appropriately handle dynamic updating of content (Blischak et al., 2016), it is rare for such mechanisms to be available for published scholarly articles. The requirement to instead publish an explicit e.g. erratum, and the rarity with which such occurs, leads to stigma against drawing excess attention to one's own errors (c.f. Strand, 2020 for an in-depth personal account). As the mechanisms for researchers to update and amend their works post-”publication” become more widely available, accepted, and utilized, for revisions both small and large in magnitude and consequence, for reasons both within and outside of authors’ control, the proliferation in dynamic revision of article content will itself de-stigmatize this process, which is necessary to uphold the mantra of scientific self-correction.

### Recommendations

From our experience we would thus like to explicitly recommend the following for both users and developers of open-source software packages in science:

• Familiarize oneself with version control systems (Blischak et al., 2016), and utilize such wherever possible.
• Construct data processing experiments in a replicable fashion (Esteban et al., 2019; Gorgolewski and Poldrack, 2016; Gorgolewski et al., 2018).
• Report in publications *precise* versions of softwares utilized, so that if issues are later discovered in such, the likelihood of their influence on experimental outcomes—and hence importance of re-processing data—can be reasonably hypothesized.
• Endeavour to engage in established software engineering practices (e.g. version control & tagging, independent code review, issue tracking, unit testing, Continuous Integration (CI) testing) as much as possible.

Finally, it is essential that research software developers and users alike engage in publicly accessible communication. This expedites the discovery and rectification of issues otherwise potentially perverting to scientific outcomes, ensures that such processes are visible and searchable to others, and dissuades evasion of accountability. This could be a software-specific forum such as community.mrtrix.org, or a more broadly-applicable but nevertheless domain-specific platform such as neurostars.org and nipy.discourse.io.

### Effect on the scientific conclusions

In the specific instance of this software bug and affected article, the negative effects were of an intermediate magnitude, such that reporting on such to the community was considered a necessity, but conversely, fundamental scientific conclusions were not altered. Further, proof of its existence was not difficult to generate using an alternative computation technique. In other instances this will not necessarily be the case: negative effects of a software bug could be:

• More subtle but nevertheless present.
• More complex and therefore more likely to evade detection.
• So substantial that they necessitate full retraction of one or even multiple manuscripts.

We assert that the comments in this article are applicable to scenarios spanning the entire spectrum of such possibilities.

## Conclusion

We have shown how one erroneous assumption in a relatively simple piece of software code can have substantial influence on another's experimental outcomes. Such issues are likely both widespread^1^ and under-reported. We hope that open exposure of the process of diagnosis, rectification and analysis replication in this instance serves both as an exemplar for the ideally bidirectional interaction between open science and research software, and as a precedent for destigmatising the broadcast of errors that may arise from this interaction; both of which are essential for the tenets of open science to transition from a theoretical aspiration to practical reality.

## CRediT authorship contribution statement

**Claude J Bajada:** Conceptualization, Methodology, Formal analysis, Writing – original draft, Writing – review & editing. **Robert E Smith:** Conceptualization, Methodology, Software, Writing – original draft, Writing – review & editing, Funding acquisition. **Svenja Caspers:** Conceptualization, Resources, Writing – review & editing, Funding acquisition.

## Data Availability

Data are available and described in the manuscript Data available at: https://db.humanconnectome.org *MRtrix3* software is available at: https://github.com/MRtrix3/mrtrix3 Results from original paper are available at: https://balsa.wustl.edu/study/1K3l Updated results are available at: https://balsa.wustl.edu/study/gmq9M

## Footnotes

1 Famously hypothesized to be 15-50 errors per 1,000 lines of code in industry (Mcconnell 2008; Soergel 2014).
